# Epidemiology of traffic injuries and motor vehicles utilization in the Capital of Iran: A population based study

**DOI:** 10.1186/1471-2458-11-488

**Published:** 2011-06-21

**Authors:** Soheil Saadat, Hamid Soori

**Affiliations:** 1 Research Assistant Professor of Epidemiology, Sina Trauma and Surgery Research Center, Tehran University of Medical Sciences, Tehran, Iran; 2 Professor of Epidemiology, Safety Promotion and Injury Prevention Research Center, Faculty of Public Health, Shahid Beheshti University of Medical Sciences, Tehran-Iran

## Abstract

**Background:**

Road traffic injuries are a serious public health problem worldwide. The incidence rate of fatal road traffic injuries is 26.4 per 100000 in the Eastern Mediterranean Region. Road traffic injuries are a major public health problem in Iran. Different routine sources are available for road traffic injuries in Iran, but they present several limitations.

This study aimed to determine the epidemiology of road traffic injuries in greater Tehran, using a population-based approach which is less prone to under-estimation compared to service-based data.

**Methods:**

In the year 2008, 2488 households were randomly selected for a face to face interview. Trained interviewers referred to the selected households to collect the subjects' demographic information, as well as their motor vehicle utilization and traffic injuries during the year prior to data collection. All interviews were recorded using a digital voice recorder and reviewed by a quality control team the day after the interview. The Student's t-test and ANOVA were used to analyze continuous variables. Chi-square test -including a test for trend for ordinal data- was used to analyze categorical variables. Ninety-five percent confidence interval was calculated for point estimates of incidences using Poisson or binomial distribution assumptions accordingly.

**Results:**

There were 119 traffic injury cases including 3 deaths (33 per 100 000) in the survey sample (n = 9100). The annual incidence of all traffic injuries for 1000 population was 13.1 (95% CI: 10.8 - 15.6), and that of fatal traffic injuries was 33.0 per 100 000 population (95% CI: 6.80 - 96.32). The annual incidence of collision traffic injury for 1000 motorcycles was 95.

**Conclusion:**

This population-based study demonstrates that the morbidity rate of RTIs is about ten times higher than the national figures reported by other available sources; and this can serve as an important warning to countries like Iran to prioritize this issue in their public health activities. To ensure more safety on our roads, we need to establish an injury surveillance system, and a more accurate national data capture system on RTIs.

## Background

Road traffic injuries (RTIs) are a serious public health problem worldwide. Globally, more than 1.2 million people are killed due to road traffic injuries, and 50 million others are injured each year. Over 90 percent of RTI deaths occur in low and middle income countries [1,2]. The incidence rate of fatal RTI is 26.4 per 100000 in the Eastern Mediterranean Region versus 17.4 per 100000 in the European Region and 19 per 100000 worldwide [3]. According to the World Health Organization (WHO) report, more research is needed on epidemiological pattern of RTIs in low and middle income countries to address the scope of the RTI problem and vulnerable road users [1].

Iran represents one of the greatest fatality rates from RTIs with 31.8 per 100000 in 2007 [4]. There is no reliable national injury surveillance system in Iran; therefore, great variation is observed on epidemiological measures of RTIs.

This study aimed to determine the following indices: the annual incidence rate of RTIs per 100000 population; the attributable risk of RTIs for motorcyclists; the incidence rate of RTIs per 10000 vehicles; and further it aimed to report the utilization pattern of motorcycles and personal vehicles in the capital of Iran with a population of 7.9 million, using a population based approach which is less prone to under-estimation compared to service based data.

## Methods

The study was carried out in Tehran (the capital of Iran) in the year 2008 using a cross sectional design. Using the registry of residential addresses of greater Tehran, 2488 households were randomly selected, out of the 4084203 in the registry. Data were collected using a structured face to face interview with an adult person residing in the selected residential location (Additional file 1). Interviewers referred to the selected households and asked for an adult household member to provide the following information on all household members: demographic information, a history of road traffic injuries during the year prior to the data collection date, and whether any household member owned a car or a motorcycle. The responders were also asked to report the age and sex of any car drivers and motorcycle riders in their household. A road traffic injury was defined as any injury (regardless of severity) that occurred while walking, bicycling, or riding in a vehicle due to a crash involving one or more vehicles (including bicycles) and originating or terminating on a roadway. RTIs were classified according to 3-digit coding system of the International classification of diseases (ICD-10) coded as V01 to V99 [5].

The inclusion criterion was being a resident of greater Tehran during 2005-2007. Residents of the institutional houses were excluded. Moreover, in case an informant was suspected to substance abuse (opioid, alcohol, etc.) by showing a bizarre behavior, he/she was excluded.

If an eligible person was not found at home or he/she declined to participate in the study, the household was skipped.

The minimum needed sample size was estimated using the following formula:

; while "p" was the proportion of RTI in Iran based on the prior published data that was 1% [1], q was (1-p), d was the precision of estimated prevalence of RTI that was set to 0.0025 and α was set to 0.05. The design effect was assumed to be 1.5; therefore, the estimated n was multiplied by 1.5.

Interviewers consisted of six health researchers who were trained for this study. A detailed protocol was provided for the interviewers instructing them how to approach the subjects, to explain the aim of the study, to ask every single question, and to provide clarification of questions using a standardized script, and also to handle potential problems. All interviews were recorded using a digital voice recorder and were reviewed by a quality control team at the day after the interview. In case of any deviance from the protocol, interviewers were promptly warned.

A double data entry was arranged by different operators, and any discrepancies in the digital data were resolved by referring to paper records.

STATA software (version 8) was used to analyze data. The Student's t-test and ANOVA were used to analyze continuous variables; Chi-square test -including a test for trend for ordinal data with more than two categories- was used to analyze categorical variables. Ninety-five percent confidence interval (CI) was calculated for point estimates of incidences using Poisson or binomial distribution assumptions accordingly.

The study protocol was approved by ethical committee of Sina Trauma Research Centre affiliated to Tehran University of Medical Sciences.

The recommendations of "the strengthening the reporting of observational studies in epidemiology (STROBE) Initiative" [6] were followed while reporting this survey.

## Results

In this survey, 2488 households consisting of 9100 individuals (males and females) were studied. There were 119 traffic injury cases including 3 deaths in the survey sample. Of all, 56 (47.1%) were motorcycle riders, 48 (40.3%) pedestrians and 15 (12.6%) car occupants (Table 1). The annual incidence of all traffic injuries for 1000 population was 13.1 (95% CI: 10.8 - 15.6) and that of fatal traffic injuries was 33.0 per 100000 population (95% CI: 6.8 - 96.3).

**Table 1:** The frequency (%) and type of road traffic injuries in different road users, during the year prior to study date

Injury	Car occupant	Motorcycle rider	Pedestrian	Total
Superficial	10 (20.0)	29 (58.0)	11 (22.0)	**50**
Open wound	3 (25.0)	8 (66.7)	1 (8.3)	**12**
Fracture	1 (2.8)	11 (30.6)	24 (66.7)	**36**
Head Injury	1 (20.0)	3 (60.0)	1 (20.0)	**5**
Tendon injury	0 (0.0)	3 (37.5)	5 (62.5)	**8**
Dislocation	0 (0.0)	1 (33.3)	2 (66.7)	**3**
Meniscus	0 (0.0)	0 (0.0)	2 (100.0)	**2**
Death	0 (0.0)	1 (33.3)	2 (66.7)	**3**

Total	15 (12.6)	56 (47.1)	48 (40.3)	**119**

Motorcycles were involved in 41.7% (n = 20) of the traffic injuries that occurred for pedestrians. Therefore, the attributable risk of RTI for motorcycles is estimated as: 47.1% _(Motorcyclists' RTI) _+ (41.7% × 40.3% _(Pedestrians' RTI)_) = 63.9%.

Seventy seven percent of pedestrians who reported a traffic injury were male (n = 37) and their mean age was 35.7 ± 16.8. There was not a statistically significant difference in age and gender of pedestrians who collided with a car compared to pedestrians who collided with a motorcycle.

Four hundred and ninety one households (19.7%) had at least one motorcycle. The total number of motorcycles was 582, and 345 (59.3%) of them belonged to the head of the household. Only 14 (2.4%) motorcycles belonged to women. The reported number of people who used a motorcycle (either owner or non-owner) was 951; of whom 594 (62.5%) drove a motorcycle, and 357 (37.5%) rode as a passenger. Women mostly used motorcycles as passengers rather than driving them (Table 2). The mean age of the passengers was 28.5 ± 16.1, while the mean age of the drivers was 35.5 ± 13.0; the difference was statistically significant (P < 0.001). The annual incidence of collision traffic injury for 1000 motorcycle was 95 (Table 3).

**Table 2:** Pattern of motorcycle riding according to gender

***Riding type***	***Male***	***Female***	***Total***
**Driver**	574 (96.6)	20 (3.4)	**594 (100)**
**Pillion**	142 (39.8)	215 (60.2)	**357 (100)**

**Total**	**716 (75.2)**	**235 (24.7)**	**951 (100)**

Pearson Chi2 = 387.5, P value < 0.001

**Table 3:** Utilization of vehicles and related RTI incidence per 1000 population (95% CI)

***Indices***		***Vehicle type***	
		Motorcycle	Car
Percentage of households having a vehicle		19.7 (18.2 - 21.4)	58.5 (56.5 - 60.4)
Vehicles/1000 population		64 (59.0-69.2)	197 (189.3-205.8)
Collision traffic injury for vehicle occupant/1000 vehicle		95 (71.2 - 123.0)	10 (5.9-15.8)
Non-collision transport accident related injury for vehicle occupant/1000 vehicle		385 (336.1-438.7)	0 (0.0-2.1)
Age of drivers	Owner	37.8 (12.94) ^1,3^	46.0 (13.29) ^1,4^
	Non-owner	28.0 (10.86) ^2,3^	32.3 (13.18) ^2,4^

^1^: P < 0.001 for age difference of car owners and motorcycle owners

^2^: P < 0.05 for age difference of non-owner car drivers versus non-owner motorcycle drivers

^3^: P < 0.001 for age difference of motorcycle owner versus non-owner drivers

^4^: P < 0.001 for age difference of car owner driver versus non-owner drivers

**Figure 1:**
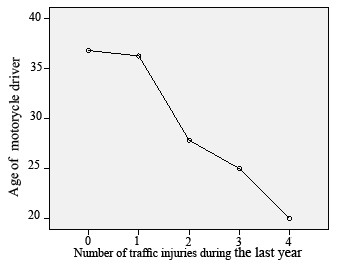
**Association of age of motorcycle drivers with the number of injurious traffic accidents during the last year**.

The age of motorcycle riders was inversely associated with the number of injurious traffic collisions (Figure 1); however, the association was not statistically significant (P-Value: 0.206).

Overall, 19.7% and 58.5% of households owned at least one motorcycle or a car respectively (Table 3).

Motorcycle drivers had experienced 193 (32.5%) non collision traffic injury (i.e. fall), while passengers had experienced 31 (8.7%) during the study period (P < 0.001). The number of injurious non collision transport accidents for motorcycle drivers was inversely related to their age (P < 0.01).

Overall, 1455 households (58.5%) had at least one car (table 3). The total number of cars was 1797; 71.1% of which belonged to the head of the household; and 219 (12.3%) belonged to women. The reported number of people who drove a car was 1763; of whom, 1515 owned a car and 248 did not own any, but drove another person's car. Female car owners were younger (39.6 years ±13.2) than male car owners (44.8 years ± 14.1) and the difference was statistically significant (P < 0.001).

Of the female car owners, 4.6% reported injurious traffic collisions during the study period compared to 2.6% of the male car owners, but the difference was not statistically significant (P > 0.05).

The mean age of car drivers reporting injurious traffic collisions was 43.9 ± 15.7 and that of drivers with no experience of injurious traffic collisions during the study period was 44.8 ± 14.0; the difference was not statistically significant.

The annual incidence of car occupant traffic injury per 1000 car was 10 (table 3).

## Discussion

This is the first published population based study indicating the incidence of traffic injuries and the utilization of motor vehicles in the capital city of Iran. This study found that morbidly rates of RTIs in greater Tehran are high and the rate of road traffic injuries is about ten times higher than the national figures reported by other sources [1,3,7,8]. However, death rate from RTIs in this study was similar to the rates reported by other sources [1]. Most published studies in this field are service based and they under-estimate the true extent of RTI in the community as they do not reflect minor injuries. In this study, we collected all traffic injuries which were severe enough to be recalled at the end of a calendar period, whether they resulted in seeking medical care or not. Superficial injuries made about 43% of the non fatal traffic injuries and were not expected to be detected in a service based surveillance system. We also noted the significant role of motorcycles in the incidence of RTIs both to riders and to the pedestrians as it was more than prior reports. The results of this study are generalizable to other big cities in Iran.

Injuries and particularly RTIs are a major public health problem in Iran. According to a study on the burden of disease and injury in Iran, 28% of disability-adjusted life years (DALYs) lost at the national level are due to injuries [9]. The RTI problem is increasing at a fast rate in developing countries due to rapid motorization [10]. This can serve as an important warning to countries comparable to Iran to prioritize this issue among their public health activities. There was a dramatic increase (69.9%) in the number of motorized vehicles in Iran between 2004 and 2007 [4] which was accompanied by an increase in road traffic crashes and an increase in the severity of injuries incurred. At the same time, public transportation has shown no significant improvement over the last decade (1997 through 2006) and accounts for only 7.1% of total journeys made [11].

### Motorcycles

In our study, 63.9% of all reported RTIs were attributable to motorcycles. Motorcycle riders constituted 47.1% of non fatal injury cases. This is more than prior reports that were mainly hospital based [12]. The collision traffic injury for motorcycles occupants was 10 folds more than car occupants. This may be explained by un-safe driving behavior of motorcycle drivers.

Although there are regulations for motorcycle riding in Iran, the law is not enforced as strongly on motorcycle riders compared to car drivers. It is not unusual for a motorcycle driver to ignore the red light or drive in the opposite direction of the traffic, and the police officers usually appear tolerant of their behaviors. Moreover, motorcycle drivers were mostly male and younger than car drivers in this study. Young age and male gender are cited as risk factors for risky driving [13]. The young age was appeared to be a risk factor for a traffic crash for motorcycle drivers in our study as well. The fact that motorcycle riders have less physical protection compared to car drivers explains another proportion of higher risk of traffic crashes [1].

Motorcycles were more often driven by a person other than their owner compared to cars. This may be another reason for motorcyclists to drive riskier than car drivers. It could be expected that the owners represent more responsible traffic behavior compared to non-owners as in Iran the police issues traffic tickets to the vehicle and not to the driver; and the owner is the one who has to pay. Driving motorcycles by people other than their owners reflects the moderate control of motorcycle owners on their vehicles (to be used by a family member for example) compared to cars, and this could be the result of police tolerance with offender motorcyclists.

Finally, motorcycle riders are at a higher risk for both collision traffic injuries as well as non-collision transport accidents that is not the case with other vehicles. In fact, for motorcycle riders, non-collision transport accident related injuries were four times more common than collision transport accident injuries (Table 3). This type of traffic accident was more common for motorcycle drivers rather than passengers and the reason may be explained by longer time of motorcycle driving compared to pillion riding.

While only 19.7% of the households had a motorcycle (compared to 58.5% that had a car), motorcycles were involved in 41.7% of the traffic injuries that had occurred amongst pedestrians. Due to lack of law enforcement, motorcycle riders may enter into areas that are designed to be used by pedestrians only, whilst this is not possible for other vehicles. Combination of high velocity of motorcycles and lack of effective separation policies from pedestrians could lead to this pattern [1,8]. Most of newly manufactured motorcycles in Iran benefit from having an engine of 125 cc size that could easily increase the speed of the motorcycle to more than 100 km/h. Heavy traffic itself limits the driving speed of cars in Tehran but motorcycles could find their track between cars and drive at high speed or simply bypass the heavy traffic by entering sidewalks; these behaviors greatly endangers the pedestrians.

### Gender

As in other countries [14-16], young males were the predominant demographic group among pedestrians who reported a traffic injury.

Less than one out of six cars belonged to women. This may reflect the income inequality between genders. In Iran, fathers are responsible for the family income. As a result, the majority of job opportunities are occupied by men. Therefore, women have limited job opportunities especially for high income jobs. However, the exceptions are the jobs that need academic education which provide fair opportunity for educated people regardless of gender. In this regard, it should be mentioned that in recent years, females constituted more than half of university students in Iran. Therefore, women in such positions are young; this is why the mean age of female car owners was less than males. However, as the majority of people (including women) do not have academic education, women generally have less access to jobs with high income; and therefore, they are less likely to afford a car.

While there are reports of higher RTC rates among male drivers as compared to female drivers [13], other studies reported a higher risk of experiencing a fatal crash for men and higher rates of involvement in injury crashes and all police-reported crashes for women [13]. We did not detect a significant association between gender and traffic crash.

In our study, women mostly rode a motorcycle as a passenger rather than a driver, and the mean age of the passengers was less than the drivers. This may reflect the fact that as an alternative to poor public transport, the motorcyclists use their motorcycle to transport their wives and children.

#### Recommendations

In 2005, the obligatory use of seat belts in Iran was initiated. Authorities now need to work on legislating and enforcing the use of other protective equipments such as child passenger restraint systems, booster seats, seat belts for rear passengers and a rear seating position for children in vehicles.

It is recommended that the effective interventions be implemented in the long term and the Iranian traffic officials/police continue to enforce the legislation accompanied by campaigns to increase public awareness about the risks and the reasons behind such legislation. Moreover, there is a need for more strict regulations and continuously serious enforcement to prevent offender motorcyclists to harm themselves and other vulnerable road users.

Some serious traffic offences should be treated as "criminal offences", especially for motorcyclists. Effective speed limit measures are needed, especially for motorcycles, either passive (i.e. stop manufacturing high speed motorcycles) or active (i.e. police enforcement).

Police needs to issue traffic tickets to drivers rather than owners of the vehicles. However, to ensure more safety on roads of Iran, there is a need for a reliable and sensitive national data capture system on RTIs and an injury surveillance system as recommended by the WHO [17,18] making it possible to identify the risk factors and to maximize the efficient use of scarce resources in targeting road safety interventions where they can have optimal impact.

#### Limitations

There were some limitations in this study. The data collected on the numbers of road traffic collisions and consequent injuries were self-reported by the study subjects. We did not verify them using another source of data, though there was no reason for responders to knowingly provide false information. We did not collect the health economic data for subjects who reported an injury. Households who declined to participate in the study (n = 23) may presented different incidence rate of morbidity and mortality of traffic injuries and this may have introduced a selection bias; however, the extent of this bias is not expected to be high considering the number of this group. The cross-section design and lack of clinical data on the severity of the RTIs are among other weaknesses of the study.

## Conclusion

The true incidence of RTI in Iran is higher than rates detected by service based surveillance systems. There is a need for reliable surveillance system to monitor RTI in Iran and similar countries.

There is a need for more rigid enforcement of traffic regulations in case of motorcycles for their own protection and also to protect other vulnerable road users.

A reliable and affordable public transport system is needed to stop people from using un-safe alternatives such as motorcycles for urban transportation.

## Abbreviations

DALY: disability adjusted life years; ICD-10: International Classification of Diseases and related health problems tenth version; USD: United States Dollar.

## Competing interests

The authors declare that they have no competing interests.

## Authors' contributions

SS designed the study, managed data collection, analyzed and composed the manuscript draft and edited the final manuscript. HS contributed to the manuscript draft and edited the final version. Both authors read and approved the final manuscript.

## Pre-publication history

The pre-publication history for this paper can be accessed here:

http://www.biomedcentral.com/1471-2458/11/488/prepub

## Supplementary Material

Additional file 1 **Data collection form**. The data collection form used by interviewers. Click here for file
