# Supplementary material for: Epidemiology of traffic injuries and motor vehicles utilization in the Capital of Iran: A population based study
Source: BMC Public Health. 2011 Jun 21;11:488. doi: 10.1186/1471-2458-11-488 (PMC3141457; doi:10.1186/1471-2458-11-488)
Supplement: Additional file 1 — Data collection form. The data collection form used by interviewers. [file 1471-2458-11-488-S1.DOC]

**Appendix 1: Data collection form**

| Cluster code | | | Household code | |
| --- | --- | --- | --- | --- |
|  |  |  |  |  |


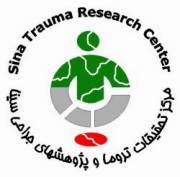


Please fill in the household members’ information

| Row number | Name | Age (years) | Relation to household head | gender |
| --- | --- | --- | --- | --- |
| 1 |  | |  |  | year | | --- | --- | --- | |  | □1. Male □ 2. female |
| 2 |  | |  |  | year | | --- | --- | --- | |  | □1. Male □ 2. female |
| 3 |  | |  |  | year | | --- | --- | --- | |  | □1. Male □ 2. female |
| 4 |  | |  |  | year | | --- | --- | --- | |  | □1. Male □ 2. female |
| 5 |  | |  |  | year | | --- | --- | --- | |  | □1. Male □ 2. female |
| 6 |  | |  |  | year | | --- | --- | --- | |  | □1. Male □ 2. female |

1. **PEDESTRIAN**

Among the household members, who has been stricken by a motor vehicle as a pedestrian during the past year?

1. I. Row number: --- When? -------- ⭘By car ⭘By motorcycle
   II. How it happened? ……………………………………………………………………………..
   III. Did he/she injured? ⭘No ⭘Yes

IV. Please describe the injury, if any :……………………………………………………………

V. Please describe the injured body region, if any :……………………………………………..............

1. I. Row number: --- When? -------- ⭘By car ⭘By motorcycle
   II. How it happened? ……………………………………………………………………………..
   III. Did he/she injured? ⭘No ⭘Yes

IV. Please describe the injury, if any :……………………………………………………………

V. Please describe the injured body region, if any :……………………………………………..............

1. I. Row number: --- When? -------- ⭘By car ⭘By motorcycle
   II. How it happened? ……………………………………………………………………………..
   III. Did he/she injured? ⭘No ⭘Yes

IV. Please describe the injury, if any :……………………………………………………………

V. Please describe the injured body region, if any :……………………………………………..............

1. I. Row number: --- When? -------- ⭘By car ⭘By motorcycle
   II. How it happened? ……………………………………………………………………………..
   III. Did he/she injured? ⭘No ⭘Yes

IV. Please describe the injury, if any :……………………………………………………………

V. Please describe the injured body region, if any :……………………………………………..............

1. I. Row number: --- When? -------- ⭘By car ⭘By motorcycle
   II. How it happened? ……………………………………………………………………………..
   III. Did he/she injured? ⭘No ⭘Yes

IV. Please describe the injury, if any :……………………………………………………………

V. Please describe the injured body region, if any :……………………………………………..............

**B. MOTORCYCLE RIDER**

Among the household members, who has been using motorcycle during the past 12 months?

1. I. Row number: --- ⭘As driver ⭘As pillion rider
   II. How many times he/she fell and injured as a non-collision traffic accident? ………………..
   III. Please describe the injury:……..……………………………………………………………..

IV. Please describe the injured body region:……………………………………………...............

V. How many times he/she had experienced a collision traffic accident? ………………..
VI. Please describe the injury :……..……………………………………………………………..

VII. Please describe the injured body region :……………………………………………...............

1. I. Row number: --- ⭘As driver ⭘As pillion rider
   II. How many times he/she fell and injured as a non-collision traffic accident? ………………..
   III. Please describe the injury :……..……………………………………………………………..

IV. Please describe the injured body region :……………………………………………...............

V. How many times he/she had experienced a collision traffic accident? ………………..
VI. Please describe the injury :……..……………………………………………………………..

VII. Please describe the injured body region :……………………………………………...............

1. I. Row number: --- ⭘As driver ⭘As pillion rider
   II. How many times he/she fell and injured as a non-collision traffic accident? ………………..
   III. Please describe the injury :……..……………………………………………………………..

IV. Please describe the injured body region :……………………………………………...............

V. How many times he/she had experienced a collision traffic accident? ………………..
VI. Please describe the injury :……..……………………………………………………………..

VII. Please describe the injured body region :……………………………………………...............

**C. CAR DRIVER**

Among the household members, who has been driving car during the past 12 months?

1. I. Row number: --- ⭘Non owner ⭘ Owner 🡪 how many cars does he/she own?.....
   II. How many traffic crashes had experienced during the past year*****? ..........
   III. Please describe the crashes: ………………………………………………………………...

IV. Please describe the injuries: ……………….………………………………………………...

V. Please describe the injured body region: ……………………………………………………...

1. I. Row number: --- ⭘Non owner ⭘ Owner 🡪 how many cars does he/she own?.....
   II. How many traffic crashes had experienced during the past year*****? ..........
   III. Please describe the crashes: ………………………………………………………………...

IV. Please describe the injuries : ……………….………………………………………………...

V. Please describe the injured body region: ……………………………………………………...

1. I. Row number: --- ⭘Non owner ⭘ Owner 🡪 how many cars does he/she own?.....
   II. How many traffic crashes had experienced during the past year*****? ..........
   III. Please describe the crashes: ………………………………………………………………...

IV. Please describe the injuries : ……………….………………………………………………...

V. Please describe the injured body region: ……………………………………………………...

* with any road user or stationary objects

**D. CAR OCCUPANT**

Among the household members, who has been injured in a traffic crash***** as a car occupant?

* with any road user or stationary objects

1. I. How many times during the past year*****? ..........
   II. Please describe the crashes: ………………………………………………………………...

III. Please describe the injuries : ……………….………………………………………………...

IV. Please describe the injured body region: ……………………………………………………...
